# Clinical outcomes of high-frequency electrocautery surgery and proton pump inhibitor therapy for refractory laryngeal contact granuloma

**DOI:** 10.1016/j.bjorl.2025.101690

**Published:** 2025-07-29

**Authors:** Zelong Wang, Xianbin Lan, Yuan Fang, Shaoping Peng, Riqun Jin, Dongming Deng, Zhiheng Song

**Affiliations:** First Affiliated Hospital of Gannan Medical University, Department of Otolaryngology, Head and Neck Surgery, Jiangxi, China

**Keywords:** Refractory laryngeal contact granuloma, Electronic laryngoscope, High-frequency electric snare, Surgical treatment, Acid suppression therapy

## Abstract

**Objective:**

To evaluate the clinical efficacy of high-frequency electrocautery under electronic laryngoscopy combined with acid-suppressing drugs (Proton Pump Inhibitor, PPI) in the treatment of Refractory Laryngeal Contact Granuloma (RLCG).

**Methods:**

A total of 42 patients with RLCG, treated at our hospital from January 2019 to January 2024 were included in this study. The patients underwent high-frequency electrocautery to excise laryngeal granulomas under local anesthesia using electronic laryngoscopy, followed by PPI therapy. Follow-up started one-month post-surgery and lasted for 6–12 months (average of 8.5 months), to assess the cure rate and overall efficacy.

**Results:**

The cure rate was 76.19%, and the overall efficacy rate was 92.86%. On average, each patient underwent 1.79 procedures. The combined treatment of high-frequency electrocautery under local anesthesia with electronic laryngoscopy and PPI therapy demonstrated significant therapeutic effects for RLCG. The treatment was simple, well-tolerated by patients, cost-effective, and resulted in rapid symptom improvement, all without the need for general anesthesia or intubation.

**Conclusion:**

The combined approach of high-frequency electrocautery under local anesthesia with electronic laryngoscopy and PPI therapy is effective in treating refractory laryngeal contact granuloma. This method offers the advantages of a straightforward surgical procedures, high patient tolerance, low cost, quick symptom relief, and no requirement for general anesthesia or intubation.

**Level of evidence:**

Level 4-Oxford Centre for Evidence-Based Medicine 2011 Levels of Evidence.

## Highlights

• Local anesthesia electrocautery via laryngoscopy treats RLCG.
• Combining with acid-suppressing therapy boosts efficacy.
• This combination shortens disease course and is highly effective.
• The procedure is simple, well-tolerated, and cost-effective.
• It improves symptoms quickly without general anesthesia or intubation.

## Introduction

Laryngeal Contact Granuloma (LCG) is a common benign proliferative lesion of the larynx, typically occurring around the vocal process in the posterior one-third of the vocal cords, either unilaterally or bilaterally. The pathogenesis of LCG is not yet fully understood, and its etiology is complex, with a high propensity for recurrence and refractoriness.^1^^,^^2^ The main symptoms of this condition include a foreign body sensation in the hypopharynx, hoarseness, frequent throat clearing, sore throat, and a burning sensation in the throat accompanied by acid reflux. Despite the availability of various treatment methods for LCG, clinical reports indicate that both medical conservative therapy and surgical treatment alone have high recurrence rates. In clinical practice, many scholars define patients whose symptoms do not improve after three months of standardized treatment, or who relapse after cure, as having Refractory Laryngeal Contact Granuloma (RLCG).^3^ The treatment of RLCG has become one of the challenging issues in otorhinolaryngology. In recent years, it has been suggested that combined treatment methods are more effective for RLCG.^4^^,^^5^ Against this background, our hospital conducted a study from January 2019 to January 2024, in which 42 patients with RLCG underwent high-frequency electrocautery surgery for laryngeal granuloma removal under local anesthesia via electronic laryngoscopy, combined with acid-suppressing drug therapy. The treatment outcomes were satisfactory.

## Methods

### General information

A total of 42 patients with RLCG treated in our department from January 2019 to January 2024 were selected as the study subjects, including 30 males and 12 females, aged 28–65 years, with an average age of 40.6 years. The disease duration ranged from 3 months to 1.5 years, with an average of 7.8 months. All patients presented with varying degrees of hoarseness and a foreign body sensation in the throat. Among them, 30 patients had frequent throat clearing, 12 had sore throat, 8 had significant acid reflux with a burning sensation, and 3 had difficulty breathing after physical activity. There were 40 cases of unilateral involvement and 2 cases of bilateral involvement. Four patients had previously undergone one session of laryngopharyngeal surgery under general anesthesia. Inclusion criteria: (1) Patients previously diagnosed with LCG who showed no significant improvement after more than three months of standardized treatment or had recurrence after cure.^3^ (2) Postoperative pathology confirmed as LCG. Exclusion criteria: (1) Patients with severe hyperglycemia, hypertension, cardiovascular disease, or other serious conditions. (2) Patients in the acute inflammatory phase, with mental illness, or allergic to local anesthetics. (3) Patients with severe dyspnea or bleeding risk. The study was reviewed and approved by the hospital's ethics committee, and all patients signed informed consent forms.

### Surgery procedure and follow-up

**Fig. 1:**
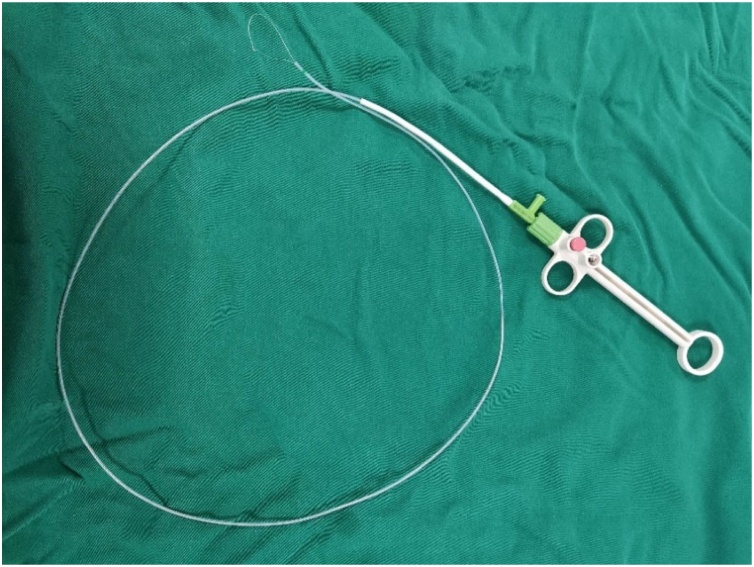
OLYMPUS 6 ZK disposable high-frequency electrocautery loop.

The observation group underwent high-frequency electrocautery to remove granulomas under local anesthesia with electronic laryngoscopy in the outpatient department. The surgical equipment included an OLYMPUS-Q290 electronic nasopharyngolaryngoscope, an ERBE VIO 200D endoscopic argon workstation, and an OLYMPUS 6 ZK disposable high-frequency electrocautery loop (Fig. 1). Before the surgery, patients received three applications of 1% tetracaine spray to the nasal cavity and laryngopharynx, with a 5-minute interval between each application. After anesthesia, patients were placed in the supine position with the head extended backward. They were instructed to breathe slowly and calmly to avoid coughing. The surgeon inserted the electronic laryngoscope through the patient's nostril to clearly visualize the laryngeal granuloma. An assistant then inserted the high-frequency electrocautery loop through the biopsy channel of the electronic laryngoscope. After the loop's protective sheath was exposed at the center of the visual field, the loop was slowly expanded and placed around the base of the granuloma. The loop was then tightened slowly. Once it was confirmed that the granuloma was completely encircled, the electrocautery pedal was pressed for 1–2 seconds (Program XY AUTO CUT mode, 35 W). The granuloma was completely removed by further tightening the loop while the electrocautery was activated. The removed granuloma was sent for routine pathological examination. After surgery, patients were instructed to fast for 2 h and then start budesonide suspension nebulization at 1 mg per dose, twice daily (BID), for one week. The following day, patients began oral administration of esomeprazole magnesium enteric-coated tablets at 20 mg per dose, twice daily (BID), for at least 8 weeks. During the treatment period, patients were advised to avoid smoking, alcohol, and spicy foods and to rest their voices to prevent excessive vocal use. All patients were followed up one month after surgery for a period of 6–12 months, with an average follow-up duration of 8.5 months.

### Efficacy evaluation criteria

The size of the granuloma before and after surgery was compared using electronic laryngoscopy during follow-up visits. The therapeutic outcomes were categorized into cure, significant improvement, improvement, and no improvement. The overall efficacy rate was the sum of the cure rate, significant improvement rate, and improvement rate.^6^^,^^7^ Cure was defined as complete disappearance of the granuloma. Significant improvement was defined as a reduction in granuloma size by more than 50%. Improvement was defined as a reduction in granuloma size between 20% and 50%. No improvement was defined as a reduction in granuloma size of less than 20% or an increase in size.^4^

## Results

The pathological examination results of all postoperative specimens showed squamous epithelium covering the mucosa, with inflammatory cell infiltration and granuloma formation, without atypical cells, which is consistent with the pathological presentation of granuloma. Within one week after surgery, symptoms of hoarseness and a foreign body sensation in the throat were significantly alleviated in all 42 patients. Among them, 25 patients (83.33%) with frequent throat clearing showed improvement, 11 patients (91.67%) with sore throat experienced symptom relief, 7 patients (87.5%) with acid reflux and a burning sensation had symptom relief, and all 3 patients with difficulty breathing after physical activity had immediate symptom relief. In the 42 patients followed up with electronic laryngoscopy, 32 patients (76.19%) were cured, 4 patients (9.25%) showed significant improvement, 3 patients (7.14%) showed improvement, and 3 patients (7.14%) showed no improvement, with an overall efficacy rate of 92.86% (39/42).

**Fig. 2:**
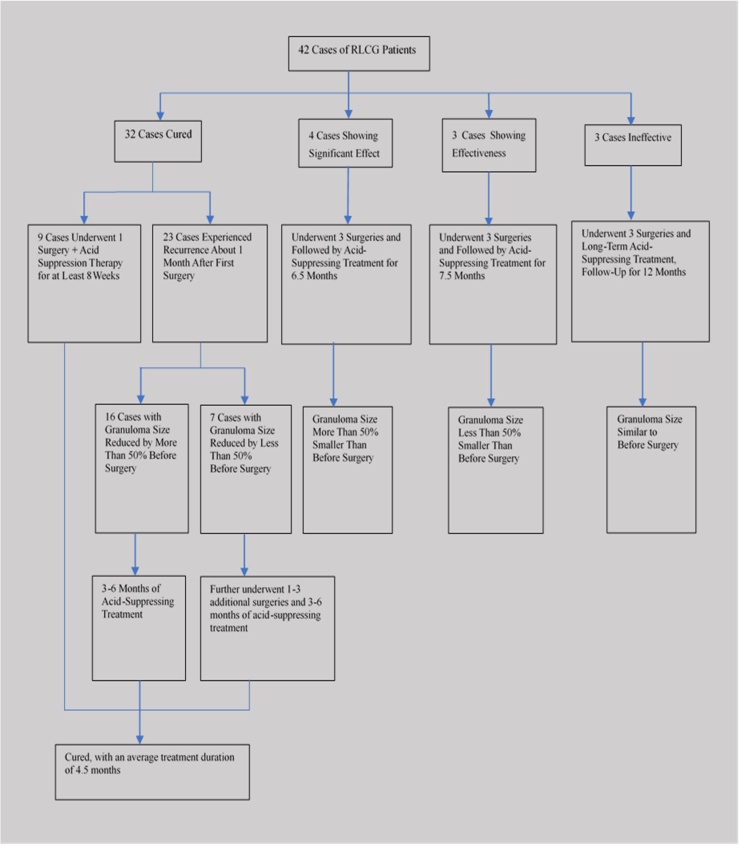
Treatment and outcome process of 42 patients with RLCG.

The specific treatment process and outcomes for the 42 patients with RLCG are as follows (Fig. 2): After 12-months of follow-up, 32 out of 42 RLCG patients were cured. Among the 32 cured patients, 9 patients underwent one surgery and acid suppression therapy, with no recurrence of the tumor observed during follow-up of more than 3–6 months. Meanwhile, 23 patients experienced recurrence around 1-month post-surgery during follow-up. Among these 23 patients, 16 patients had a reduction in granuloma size of more than 50% compared to preoperatively. These 16 patients were cured after continued acid-suppressing therapy for 3–6 months. The remaining 7 patients had a reduction in granuloma size of less than 50% compared to preoperatively and underwent 1–3 additional surgeries, followed by acid-suppressing therapy for 3–6 months, resulting in cure. The average cure time for the 32 cured patients was 4.5 months. Among the 42 patients with RLCG, 4 patients had a reduction in granuloma size of more than 50% compared to pre-treatment, with an average of 3 surgeries per patient and an average duration of acid-suppressing therapy of 6.5-months. Three patients had a reduction in granuloma size of less than 50% compared to pre-treatment, all underwent 3 surgeries, and had an average duration of acid-suppressing therapy of 7.5-months. Another 3 patients underwent 3 surgeries combined with acid-suppressing therapy and vocal rest, and after 12-months of follow-up, the size of the granuloma remained similar to pre-treatment. The average number of surgeries per patient in this group was 1.79, and no postoperative complications or adverse drug reactions were observed.

**Fig. 3:**
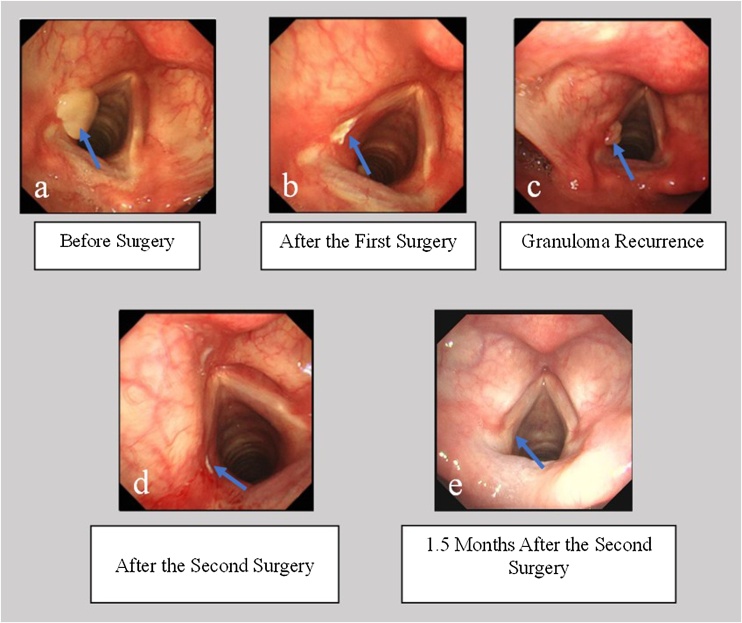
(a) A 48-year-old male with refractory granuloma on the left vocal cord. The arrow indicates the granuloma. (b) Image after the first surgery, with the arrow pointing to the surgical wound. (c) One and a half months after the first surgery, the recurrent granuloma appeared again. The arrow indicates the recurrent granuloma. (d) Image after the second surgery, with the arrow pointing to the surgical wound. (e) One and a half months after the second surgery, the granuloma completely disappeared. The arrow indicates the original site of the granuloma.

Fig. 3 shows the treatment of a 48-year-old male with left vocal cord refractory granuloma. High-frequency electrocautery was performed under local anesthesia with electronic laryngoscopy to remove the granuloma, followed by acid-suppressing treatment and vocal rest. One and a half months after the first surgery, electronic laryngoscopy revealed recurrence of the granuloma, with a size reduction of more than 50% compared to preoperatively. The patient was treated again with surgery combined with acid-suppressing therapy and vocal rest. One and a half months after the second surgery, the granuloma completely disappeared, and the pathology after both surgeries confirmed granuloma.

## Discussion

LCG is not uncommon in clinical practice and is challenging to treat due to its tendency to recur. Pathologically, repeated physical and chemical irritation causes mucosal damage around the vocal process, leading to inflammatory cell infiltration and the formation of granulation tissue. The etiology is complex, and the main causes currently recognized include tracheal intubation trauma, excessive vocal use, and laryngopharyngeal reflux.^8^ Various treatment methods are available for LCG, including acid-suppressing drug therapy, voice therapy, vocal rest, injection of botulinum toxin type A into the laryngeal muscles, injection of corticosteroids into the granuloma, and various surgical procedures. Due to the significant role of laryngopharyngeal reflux in the pathogenesis of LCG, acid-suppressing therapy is widely recommended as the first-line treatment for this condition both domestically and internationally.^9^ As early as 1999, Wani et al.^10^ treated 21 patients with LCG with acid-suppressing therapy combined with vocal rest, resulting in complete disappearance of granulomas in 14 patients within six months, significant reduction in four patients, and an overall efficacy rate of 85.7%. This is consistent with the effective rate of conservative treatment reported in recent domestic studies. Zheng et al.^11^ treated 18 patients with LCG using acid-suppressing conservative therapy, combined with avoiding excessive vocal use, correcting poor vocal habits, and lifestyle changes, achieving an effective rate of 83.3%. Wang et al.^12^ followed up 53 untreated patients with LCG for up to 70-weeks and found that 82% of the patients had complete disappearance of granulomas within an average of 30.7 weeks, indicating a certain degree of spontaneous remission of the disease. Acid-suppressing therapy and vocal rest, which prevent continuous vocal cord injury, are effective and contribute to shortening the disease course.

Currently, surgery is not recommended as the first-line treatment for LCG for two main reasons: First, the recurrence rate of surgical resection is high. Tsai et al.^13^ conducted a systematic review of the treatment of LCG and found that the recurrence rate of surgical resection was significantly higher than that of conservative treatment (29% vs. 16%). Second, surgery itself is a form of stimulation to the affected area. Surgery only removes the lesion without addressing the underlying cause of the disease and may contribute to the refractory nature of laryngeal granuloma.^14^ Pan et al.^15^ found that the total effective rate of patients with a history of surgery (71.5%) was significantly lower than that of patients without a history of surgery (97.7%) after treating 390 patients with LCG with combined injection of corticosteroids into the granuloma and acid-suppressing therapy. Therefore, it is not recommended to use surgical treatment alone as the first-line treatment option.

In fact, there is currently no unified diagnostic criterion for Refractory Laryngeal Contact Granuloma (RLCG) both domestically and internationally. In 2016, Tian et al.^3^ proposed that patients who had not healed after more than three months of one or more standardized treatments, or who had relapsed after cure, should be diagnosed with RLCG. This study also adopted this criterion as the inclusion standard for RLCG patients in this group. All 42 patients in this group were diagnosed with RLCG. Both domestic and international research on such patients have consistently shown that comprehensive treatment can achieve more significant therapeutic effects. Nie et al.^4^ compared the treatment outcomes of patients with recurrences who received either acid suppression alone or a combination of acid suppression and intralesional steroid injection. The results showed that the combination therapy had significantly higher cure rates and lower recurrence rates compared to acid suppression alone (81.20% and 57.58% vs. 3% and 12.12%, respectively). Zang et al.^16^ treated 20 RLCG patients with CO_2_ laser resection under suspension laryngoscopy combined with intralesional injection of botulinum toxin type A on the affected side, postoperative acid suppression, and vocal cord rest. The clinical symptoms of the patients improved rapidly after surgery, and the cure rate was found to be 95% after an average follow-up of 18-months. Although botulinum toxin type A, steroid injection, and surgical resection under suspension laryngoscopy have relatively solid evidence-based medical support in the comprehensive treatment of RLCG, they also have certain limitations. The injection of botulinum toxin type A and steroids requires a high level of technical skill from the operator. There is currently no unified standard for the injection dosage. Moreover, botulinum toxin injection may cause neuromuscular junction transmission disorders, leading to transient vocal cord paralysis, which can cause hoarseness, coughing while drinking, and other symptoms. Such treatments cannot quickly alleviate symptoms in patients with severe symptoms affecting respiration.^17^^,^^18^ The limitations of current surgical treatments include the need for general anesthesia and intubation, which are not suitable for patients in poor general condition who cannot tolerate surgery. Additionally, intubation under general anesthesia is itself one of the etiological factors for LCG.^2^ The surgical method used in this study was performed under local anesthesia, avoiding general anesthesia and tracheal intubation compared to conventional surgical methods. Compared to intralesional injections of botulinum toxin or steroids, it can immediately improve symptoms after surgery. For patients who cannot tolerate electronic laryngoscopy under local anesthesia, treatment options such as surgery under general anesthesia with a supported laryngoscope, local medication injection, or conservative medical therapy can be selected based on the patient's condition.^19^

In this study, we used high-frequency electrocautery to remove laryngeal granulomas under local anesthesia with electronic laryngoscopy in the outpatient department. The surgery lasted about 5−15 min, and patients quickly improved symptoms such as difficulty breathing, hoarseness, foreign body sensation in the throat, and frequent throat clearing. The average number of surgeries per patient in this group of 42 patients was 1.79. The average cost per surgery (excluding acid-suppressing drugs) was about 2,000 yuan. The cure rate was 76.19%, and the overall efficacy rate was 92.86%, which is consistent with the combined treatment effects of RLCG reported domestically and internationally. Since all patients in this group had RLCG, they had already understood the conventional treatment methods, effects, and costs of the disease before coming to our center. Therefore, the postoperative compliance of patients in this group was high. Even if they had a recurrence and needed another surgery, they could accept it because, for patients, outpatient local anesthesia surgery is time-saving, cost-effective, and rapidly improves symptoms without the need for hospitalization and general anesthesia. Since 2015, our center has gained certain experience in treating benign laryngopharyngeal tumors with high-frequency electrocautery under electronic laryngoscopy, such as epiglottic cysts, pedunculated laryngopharyngeal papillomas, and laryngeal granulomas after laryngectomy for laryngeal cancer.^20^^,^^21^

Summary of the clinical treatment experience of 42 cases in this group: (1) During surgery, the high-frequency electrocautery loop must completely encircle the granuloma at its base before tightening. The workstation should be adjusted to low-power electrocautery mode (Program XY AUTO CUT mode, 35 W). When the electrocautery pedal is pressed, the assistant should further tighten the loop until the granuloma is completely removed. This can minimize damage to the local mucosal tissue. This experience comes from our center's treatment of epiglottic cysts with high-frequency electrocautery.^20^ (2) Surgical removal of the granuloma can quickly improve patients' symptoms but does not address the underlying cause. Therefore, postoperative acid-suppressing therapy, vocal rest, and nebulization therapy should be considered as important as surgery. (3) There is currently no unified standard for the criteria for reoperation of LCG. In this study, a postoperative granuloma size greater than 50% of the preoperative size was used as the criterion for reoperation. Those with less than 50% were continued with conservative treatment. From the research results, it can be seen that the 16 patients who had a recurrence with a granuloma size less than 50% of the preoperative size were cured after 3–6-months of acid-suppressing therapy. This indirectly proves the rationality of this criterion. However, this study was conducted in a single center with a small number of cases, and further research is needed to confirm its rationality. At present, it can only be used as a reference for clinical experience. Fortunately, this technique is simple and easy to learn. Professional otolaryngologists can master it after about one month of training, which is conducive to the effective promotion of this new surgical method. This will help facilitate future multicenter studies to address the limitations of the current research. (4) Although the results of this study show that high-frequency electrocautery under local anesthesia with electronic laryngoscopy combined with acid-suppressing drugs can achieve good therapeutic effects in treating RLCG and has the advantages of simple surgical procedures, strong patient tolerance, low cost, rapid symptom improvement, and no need for general anesthesia and intubation, the number of cases is still small, and the follow-up time is short. Further verification is needed in multicenter clinical work in the future.

## Conclusion

The treatment of RLCG by high-frequency electrocautery resection of laryngeal granuloma under local anesthesia via electronic laryngoscopy, combined with acid-suppressing medications, has achieved significant therapeutic effects. Moreover, it has several advantages, including simple surgical procedures, strong patient tolerance, low cost, rapid symptom improvement, and no need for general anesthesia and endotracheal intubation.

## ORCID ID

Zelong Wang: 0009-0002-1107-6695

Yuan Fang: 0009-0002-1327-7087

Shaoping Peng: 0009-0006-9298-6621

Riqun Jin: 0009-0004-4603-3470

Dongming Deng: 0009-0006-1342-0065

Zhiheng Song: 0009-0004-7463-9568

## Ethical approval

All procedures performed in studies involving human participants were in accordance with the ethical standards of the institutional and/or national research committee and with WMA Declaration of Helsinki ‒ Ethical Principles for Medical Research Involving Human Subjects. All patients were informed of the surgical risks and signed the informed consent form before the operation.

## Funding

This research did not receive any specific grant from funding agencies in the public, commercial, or not-for-profit sectors.

## Declaration of competing interest

The authors declare no conflicts of interest.

## References

[1] Adessa M., Xiao R., Hull D. (2020). Benign vocal fold lesions in patients with chronic cough. Otolaryngol Neck Surg..

[2] Ostrander B.T., Yu V., Vahabzadeh-Hagh A. (2023). Bilateral vocal fold granuloma and anterior glottic web after papilloma excision. Ear Nose Throat J..

[3] Tian Shiyu, Li Jinrang (2015). Acid-suppressing drug therapy for laryngeal contact granuloma. Chin J Otorhinolaryngol Head Neck Surg..

[4] Nie Q., Li J., Zou S., Zhang R. (2021). Comparison of PPI and combined treatment in the treatment of recurrent laryngeal contact granuloma. Am J Otolaryngol..

[5] Yılmaz T. (2013). Recurrent contact granuloma experience with excision and botulinum toxin injectionrecurrent contact granuloma. JAMA Otolaryngol Neck Surg..

[6] Pan Yufei, Li Jinrang (2023). Study on the causes and treatment of refractory laryngeal contact. South Med Univ..

[7] Wang J.S., Li J.R., Pan Y.F., Liu Z., Zhang C., Wang W.J. (2024). Analysis of the efficacy of vocal cord botulinum toxin injection for refractory laryngeal contact granuloma. Zhonghua Er Bi Yan Hou Tou Jing Wai Ke Za Zhi..

[8] Carroll T.L., Gartner-Schmidt J., Statham M.M., Rosen C.A. (2010). Vocal process granuloma and glottal insufficiency: an overlooked etiology?. Laryngoscope..

[9] Nie Qian, Li Jinrang, Zhang Ran (2020). The impact of laryngopharyngeal reflux on the treatment efficacy of male patients with vocal process granuloma. J Audiol Speech Pathology..

[10] Wani M.K., Woodson G.E. (1999). Laryngeal contact granuloma. Laryngoscope..

[11] Zheng Meijun, Yang Hui, Lv Dan (2017). Clinical treatment analysis of 18 cases of laryngeal contact granuloma. Clin J Otorhinolaryngol Head Neck Surg..

[12] Wang C.P., Ko J.Y., Wang Y.H., Hu Y.L., Hsiao T.Y. (2009). Vocal process granuloma – A result of long-term observation in 53 patients. Oral Oncol..

[13] Tsai S.W., Ma Y.F., Shih L.C., Tsou Y.A., Sung C.K. (2021). Operative and conservative management of laryngeal contact granuloma: a network analysis and systematic review. J Voice..

[14] Liang T.J., Wang N.Y., Liu S.I., Chen I.S. (2021). Vocal cord granuloma after transoral thyroidectomy using oral endotracheal intubation: two case reports. BMC Anesthesiol..

[15] Pan Yufei, Li Jinrang, Nie Qian (2022). The impact of previous surgical treatment on idiopathic laryngeal contact granuloma. Clin J Otorhinolaryngol Head Neck Surg..

[16] Zang Yanzhi, Wan Baoluo, Wang Guangke (2018). Efficacy analysis of CO_2_ laser surgery combined with botulinum toxin type A injection in the treatment of refractory laryngeal contact granuloma. Chin J Otorhinolaryngol Head Neck Surg..

[17] Ban M.J., Ryu C.H., Korean Society of Laryngology, Phoniatrics, and Logopedics Guideline Task Force (2023). Guidelines for the use of botulinum toxin in otolaryngology from the Korean society of laryngology, phoniatrics and logopedics guideline task force. Clin Exp Otorhinolaryngol.

[18] Teng T.Z.J., Zhai C., Ng C.H.L. (2023). Vocal Fold Granuloma: Updates and Advancements in Treatment. J Voice.

[19] Tian S.Y., Li J.R. (2017). Current status of treatment of laryngeal contact granuloma. Lin Chuang Er Bi Yan Hou Tou Jing Wai Ke Za Zhi..

[20] Lan Xianbin, Peng Shaoping, Wu Guiqing (2024). Efficacy analysis of high-frequency electrocautery under electronic laryngoscopy in the treatment of epiglottic cyst. Chongqing Med J..

[21] Liu Xuemei, Zhang Jianhua, Jin Riqun (2023). Application of dexamethasone injection nebulization combined with electrocautery technique under electronic laryngoscopy in patients with inflammatory granuloma after partial laryngectomy. Chin Med Innovation..

